# *Dejian* Mind-Body Intervention Improves the Cognitive Functions of a Child with Autism

**DOI:** 10.1155/2011/549254

**Published:** 2011-03-28

**Authors:** Agnes S. Chan, Sophia L. Sze, Mei-Chun Cheung, Yvonne M. Y. Han, Winnie W. M. Leung, Dejian Shi

**Affiliations:** ^1^Neuropsychology Laboratory, Department of Psychology, The Chinese University of Hong Kong, Shatin, Hong Kong; ^2^Integrative Neuropsychological Rehabilitation Center, The Chinese University of Hong Kong, Shatin, Hong Kong; ^3^Henan Songshan Research Institute for Chanwuyi, Henan 452470, China; ^4^Institute of Textiles and Clothing, The Hong Kong Polytechnic University, Kowloon, Hong Kong; ^5^Department of Special Education and Counselling, The Hong Kong Institute of Education, Tai Po, Hong Kong

## Abstract

There has been increasing empirical evidence for the enhancing effects of *Dejian* Mind-Body Intervention (DMBI), a traditional Chinese *Shaolin* healing approach, on human frontal brain activity/functions, including patients with autism who are well documented to have frontal lobe problems. This study aims to compare the effects of DMBI with a conventional behavioural/cognitive intervention (CI) on enhancing the executive functions and memory of a nine-year-old boy with low-functioning autism (KY) and to explore possible underlying neural mechanism using EEG theta cordance. At post-one-month DMBI, KY's inhibitory control, cognitive flexibility, and memory functioning have significantly improved from “severely-to-moderately impaired” to “within-normal” range. This improvement was not observed from previous 12-month CI. Furthermore, KY showed increased cordance gradually extending from the anterior to the posterior brain region, suggesting possible neural mechanism underlying his cognitive improvement. These findings have implicated potential applicability of DMBI as a rehabilitation program for patients with severe frontal lobe and/or memory disorders.

## 1. Introduction

Autism is a neurodevelopmental disorder with clinical manifestations similar to those of frontal lobe/executive dysfunctions, including inflexible thinking, disinhibited emotional reactivity, repetitive or socially inappropriate acts. Structural and functional abnormalities in the frontal lobes of such individuals are well documented [1–4]. Conventionally, autism is remediated by minimizing environmental triggers, replacing misbehaviors with socially acceptable expression through functional communication training or behavior modification [5–7]. Effective treatments can reduce problematic behaviors up to 85% [8], and yet the treatment outcomes are context-specific and are labor- and time-intensive requiring 20–40 hours weekly therapy for two or more years [9]. Since autism is incurable and has pervasive impacts on affected individuals, several studies had explored and evidenced cost-effectiveness and efficacy of traditional Chinese medicine as complementary intervention [10–14]. These treatments shared the common philosophy of unblocking the internal bodily energy (*Qi*). Methods that facilitate the flow of *Qi* in restoring balance in the body (including the brain) are believed to be effective in improving all-round functioning in autism. 

*Dejian* Mind-Body Intervention (DMBI) was developed based upon traditional Chinese *Shaolin* healing practice (*Chanyi)* by the last and first authors. *Chanyi* is a unique healing approach to strengthen the mind power and bodily wellness by unblocking *Qi* and clearing bodily orifices (e.g., nasal cavity). DMBI comprises four interconnected components of *Chan* practice, mind-body exercises, dietary monitoring, and opening the orifices [15]. Several studies have been conducted to examine the effect of this newly developed therapeutic technique. A randomized controlled trial of a four-session DMBI significantly reduced negative moods and elevated frontal EEG alpha asymmetry of community-dwelling adults [16]. A controlled trial reported a positive change of the neural electrophysiological state after practicing Dan Tian Breathing (one of the mind-body exercises in DMBI) compared with conventional relaxation technique [17]. Another study reported the enhancement of electrophysiological activity in the frontal and anterior cingulate cortex (regions mediating inhibitory control) immediately after intranasal application of a specially formulated *Chanyi* herbal nasal drop [18]. An adolescent with Asperger's disorder showed significantly reduced repetitive behaviors and temper outbursts after a three-month DMBI [19]. In addition, significant improvement in cognitive and adaptive living abilities, physical health, and/or mood has been observed in over hundreds of clinical cases treated with the *Chanyi* approach by the last author and some other monks in the temple. These cases included patients with neurological (e.g., motor neuron disease, stroke, brain tumor) and psychiatric disorders (e.g., schizophrenia, depression), and physical health problems (e.g., low back pain, constipation) [15].

In the present study, we explored DMBI's effect on improving the cognitive functions of a child with low-functioning autism, who showed minimal improvement after one year of conventional intervention (CI). In addition, we examined the underlying possible neural mechanism associated with this improvement by using the EEG cordance measure.

## 2. Materials and Methods

### 2.1. Subject

KY is a 9-year-5-month-old right-handed boy with mental retardation and autism. He had received one year of physiotherapy at age 1.5, and five years of alternative treatment (including acupuncture, energy training and *Qigong*) since age 2, for his delayed sensory, language, motor, and social developments, with limited effect. Given KY's frequent temper tantrums (≥5 times weekly, lasting 20–60 minutes per time) and his very brief attention and memory span, his parents brought him to the Integrative Neuropsychological Rehabilitation Center (INRC) at age 7 for conventional neuropsychological intervention.

### 2.2. Neuropsychological Measures

The Hong Kong List Learning Test (HKLLT) [20], a well-established Chinese-word list learning test consisting of free recall at three learning and two delayed trials, and a recognition trial, was used to measure memory and inhibition. The Children's Color Trails Test (CCTT) [21] was also administered to assess inhibitory control and cognitive flexibility, using the errors committed in the second trial as an index. Parent's ratings on the Behaviour Rating Inventory of Executive Function (BRIEF) [22] was also administered to assess overall executive functioning (Global Executive Composite, GEC) and inhibitory control (Behavioral Regulation Index, BRI).

KY was assessed at pre- and post-12-month CI and post-1-month and post-8-month DMBI, where the post-CI also represented the pre-DMBI assessment. All assessments were administered by the clinical psychologist who did not provide the DMBI for KY. As suggested by Jacobson et al. [23], KY's pre-post performance change would be considered reliable and clinically significant if his initially impaired functioning would fall within the normal range after intervention.

### 2.3. Treatment Protocol

**Figure 1:**
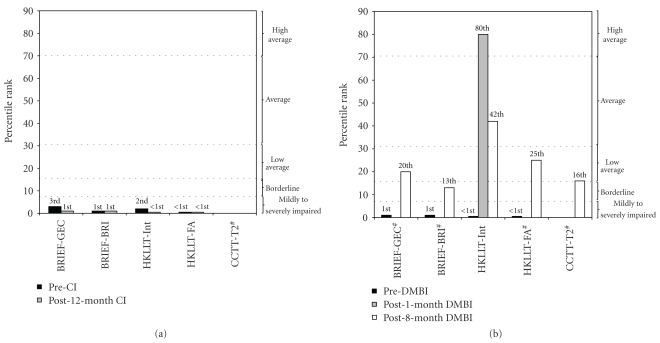
Percentile changes in executive functions of KY at (a) pre- and post-CI and at (b) pre- and post-DMBI. Post-CI and Pre-DMBI are at the same measurement time point. Higher percentile represents better performance. BRIEF: Behavior Rating Inventory of Executive Function; GEC: General Executive Composite; BRI: Behavioral Regulation Index; HKLLT: Hong Kong List Learning Test; Int: Intrusion errors; FA: False Alarm; CCTT: Children's Color Trail Test. ^#^missing bar in HKLLT and CCTT as KY failed to comprehend or finish the tasks; while missing bar in BRIEF as it was not administered at post-1-month DMBI.

**Figure 2:**
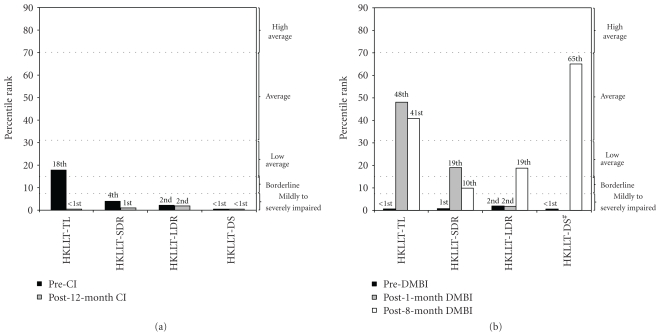
Percentile changes in memory functions of KY at (a) pre- and post-CI and at (b) pre- and post-DMBI. Post-CI and Pre-DMBI are at the same measurement time point. Higher percentile represents better performance. HKLLT: Hong Kong List Learning Test; TL: Total Learning; SDR: Short (10-min) Delayed Recall; LDR: Long (30-min) Delayed Recall; DS: Discrimination Score. ^#^missing bar in HKLLT as KY failed to finish the task.

CI. KY was trained on impulse control and attention by the second author, a clinical psychologist, for 30 minutes per week for a year. This involved functional communication training with behavioral modification techniques, substituting his temper outbursts with socially appropriate expressions (e.g., “I don't know”) and 30-minute daily home practices of a computerized attention and impulse control training program developed by the INRC. After the 12-month CI, KY's duration of temper tantrum reduced from >20 minutes to <5 minutes. However, no improvement was shown in his inhibitory control and memory functioning, which remained to be moderately to severely impaired (Figures 1(a) and 2(a)). His mother was thus introduced to the DMBI.

**DMBI**

KY was treated with DMBI by the first author for 15-minute weekly sessions during the first month, and 15-minute monthly sessions in the subsequent seven months, plus home application of three of the four integrated components in DMBI. *Chan* practice was not applicable to KY given his limited intelligence.

#### 2.3.1. Dietary Monitoring

KY was recommended to take seven categories of food everyday and with one to three kinds from each category. The seven categories are (1) grains (examples of kinds: noodles, brown rice, barley), (2) vegetables (e.g., broccolis, cabbages, tomatoes), (3) fruits (e.g., grapes, apples, oranges), (4) beans (e.g., soy, red beans, peas), (5) mushrooms (e.g., black fungus, white fungus, straw mushrooms), (6) nuts (e.g., walnuts, chestnuts, almonds), and (7) roots (e.g., taros, potatoes, yams). The amount of food was not fixed and it was recommended that the child ate up to 80% full in each meal. More importantly, KY has abstained from ginger, garlic, green onion, spicy foods, and seafood, and reduced 70% intake of meat since DMBI. These foods are believed to be hard to digest by people with illnesses and easily generate excessive heat inside the body and block the orifices. KY's dietary change was recorded by his parents on a questionnaire about the types of food taken. According to his mother's report, KY's dietary has met the daily recommended guideline during the intervention.

#### 2.3.2. Herbal Nasal Drop

KY applied intranasally a specially formulated herbal remedy at the dosage of 10 mL twice daily to clear his nasal cavity. The herbal remedy was manufactured following the product safety guidelines of the Hong Kong Department of Health for heavy metal, pesticides residue, and microbiological substances. The remedy was manufactured by the Hong Kong Institute of Biotechnology that is a Chinese Medicine Manufacturer meeting the Good Manufacturing Practice standards and is owned by the Chinese University of Hong Kong. The herbal nasal drop has been produced for research purpose only, and not for commercial purpose. The formula is currently under testing and modifying for safety, effectiveness, and patent application. Some major ingredients include Herba Artemisiae Annuae and Rhizoma Coptidis. 

#### 2.3.3. Mind-Body Exercises

KY's mother performed Nose-Bridge Massage for KY to unblock his nasal cavity, by softly and slowly moving her index fingers up and down each side of KY's nose bridge for 36 times every night. KY also practiced Tranquil Stand, that is, standing squarely and relaxingly on both feet and raising the hands gently in front of his abdomen. Instead of practising every day, KY was only willing to practice once to twice weekly, for <5 seconds at the beginning to one minute after one year of practice. 

### 2.4. Neuroelectrophysiological Measures

Quantitative EEG data were recorded from 19 electrodes based on the International 10–20 system during eyes-open resting state before and after 1-month and 8-month DMBI. The EEG signal was digitized at 256 Hz with a low pass filter of 30 Hz, and impedances below 10 kΩ. At least one minute of artefact-free data were selected and computed into cordance indices using a three-step algorithm [24].

### 2.5. Cordance Intensity

Cordance has been proposed as an indirect measure of brain perfusion, where higher perfusion implicates higher metabolism. Cordance values were grouped topographically into anterior (FP1, FP2, F3, F4, F7, F8, Fz, and Cz), centrotemporal (C3, C4, T3, T4, T5, and T6), and posterior (P3, P4, Pz, O1, and O2) regions to provide a measure of mean cordance intensity for each region. Theta (4–7 Hz) cordance intensity was used in this study given its association with cerebral perfusion [24, 25], and its deficiency in the anterior brain region being correlated with executive dysfunctions in children with autism [5].

### 2.6. Global Brain State

This represents the proportion of electrodes showing concordance (quadrant II, IV) or discordance (quadrant I, III) along the two dimensions of absolute and relative power (Figure 4). The electrode was “concordant”, when both absolute and relative power were above (Abs+ and Rel+) or below (Abs− and Rel−) the mean value for a particular site. Otherwise, it was “discordant” (Abs+ and Rel−; Abs− and Rel+). Simultaneous qEEG and PET studies revealed higher cerebral perfusion underlying concordant electrodes [24].

## 3. Results

### 3.1. DMBI Enhanced Inhibitory Control and Cognitive Flexibility

Compared with KY's severely to moderately impaired inhibitory control at post-12-month CI (Figure 1(a)), KY showed significant improvement after one month of DMBI (Figure 1(b)) with a reduction in Intrusion score from 9 to 0, as assessed by the HKLLT. At post-8-month-DMBI, his inhibitory control and cognitive flexibility improved from “severely impaired” to “low average to average” as measured by the Intrusion score (non target word) and False Alarm (new words misidentified as learned words) on the HKLLT, and set-shifting error in CCTT. His mother's rating on KY using the BRIEF improved from “moderately impaired” to “borderline (in BRI) and low average (in GEC)” at post-8-month DMBI. KY's emotional outburst frequency reduced from ≥5 times weekly to twice weekly at post-1-month DMBI. At post-8-month-DMBI, he reacted with patience, calmness, and understanding upon sudden changes and unmet demands.

### 3.2. DMBI Enhanced Learning and Memory

KY demonstrated significant memory enhancement from the “severely impaired” to the “low-average to average” level after receiving DMBI (Figure 2(b)). KY's pre-DMBI memory profile was consistent with low-functioning autism with pervasive impairment in encoding, retention and retrieval of memory [26]. After one year of CI, KY's performance in total learning (3 words), delayed recall (none) and discrimination score (8%; calculated as (Correct Hit − False Alarm)/16∗100) remained severely to moderately impaired (Figure 2(a)). At post-1-month DMBI, KY's total learning (19 words) improved to normal average level. At post-8-month-DMBI, his ability to recall (71%) and discriminate (92%) target words at 30-minute delayed recall and recognition were within low-average to average range, respectively.

### 3.3. DMBI Elevated Theta Cordance

**Figure 3:**
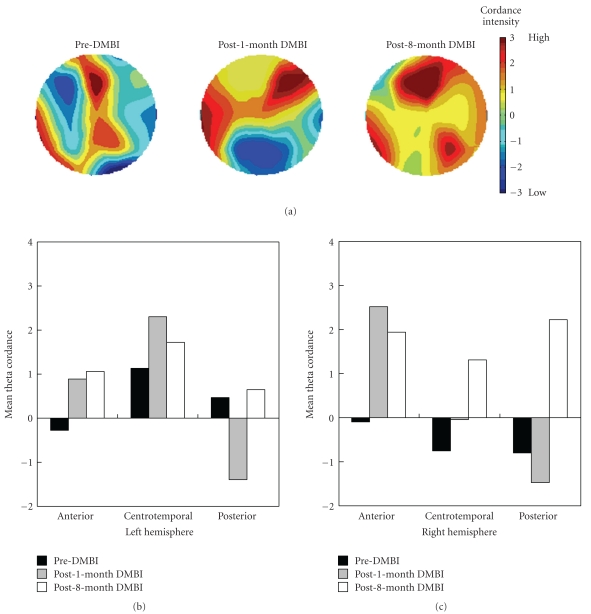
Topographic and bar illustration of changes in theta cordance intensity of KY at pre-DMBI, post-1-month DMBI, and post-8-month-DMBI. Orange-red indicates higher cordance value, and green-blue indicates lower cordance value.

At pre-DMBI, KY showed suppressed theta cordance value in the right hemisphere and bilateral anterior regions (Figure 3). At post-1-month DMBI, KY showed elevated anterior cordance to 1.55 (a double of 0.74 at pre-DMBI), which was consistent with previous findings of enhanced anterior cordance associated with the use of the herbal nasal drop [18]. At post-8-month DMBI, it increased further to 1.85 (1.5 times higher than pre-DMBI) and spread to more posterior regions. KY's right-hemispheric cordance was enhanced from −0.549 to 0.33 at post-1-month DMBI, and further to 1.82 at post-8-month DMBI.

### 3.4. DMBI Fostered Concordant Brain State

**Figure 4:**
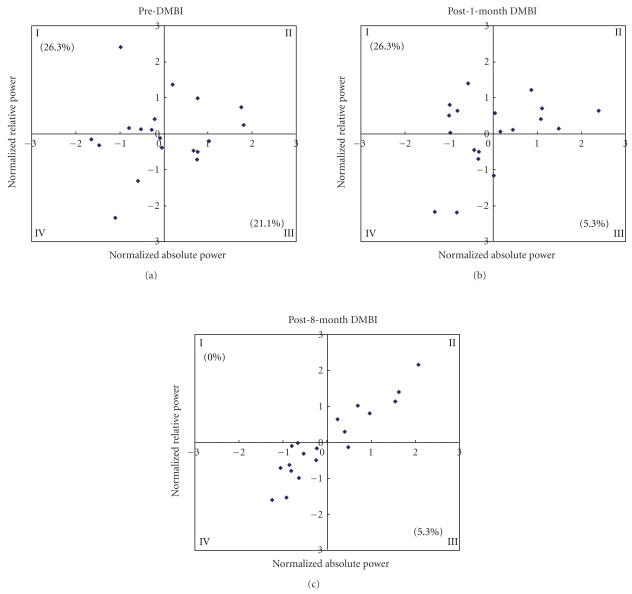
Changes in global brain state as reflected by the altered distribution of concordant and discordant electrodes spread throughout the scalp of KY at (a) pre-DMBI, (b) post-1-month DMBI, and (c) post-8-month DMBI. Each point represents the pair of normalized absolute and relative power values for each of the 19 electrodes.

Furthermore, KY's global brain state became more concordant (suggesting higher perfusion) when the intervention progressed, as indicated by more and more electrodes turned from discordant (i.e., quadrant I & III) into concordant (i.e., quadrant II & IV) state. At pre-DMBI, KY had 47% of electrodes (9 out of 19 electrodes placing in quadrant I & III) displaying discordance (Figure 4(a)), which is deviated from the normal distribution of less than one-third of discordant electrodes observed in healthy individuals. Discordant brain state has been reported to be associated with reduced cerebral perfusion and pathological change in the brain [24, 25]. At one-month and eight-month-post-DMBI, the proportion of discordant electrodes has reduced to 31% (6/19 electrodes) and 5% (1/19 electrodes), respectively (Figures 4(b) and 4(c)). 

## 4. Discussion

This study revealed the positive effects of DMBI on inhibitory control and memory of a child with low-functioning autism, from impaired level to normal level after 8-month-DMBI. Given KY's minimal improvement after 12-month CI, his enhancement at post-8-month-DMBI is unlikely to be due to maturation or spontaneous response to any treatment. In addition, the EEG measures provided insights into the possible neural mechanism that may be associated with the improvement of executive and memory functions. That is, the increased anterior cordance and the increasingly concordant global brain state at post-8-month-DMBI may suggest a change of neural activities. Given the well-documented involvement of frontal-temporal/parietal connections in attentional and memory processing [27], the increased posterior cordance might implicate KY's improvement in frontal and memory functioning.

The majority of conventional intervention for individuals with low-functioning autism is behavioral or educational based, which are time and labor intensive [9] and with relatively few effective treatments for their impaired cognitive functions. The positive effects of DMBI on this child with autism were encouraging. However, the findings need to be further examined in larger samples in randomized controlled trials.

## 5. Conclusion

The present study has provided encouraging findings on the potential effects of DMBI on improving the cognitive functions and altering the neural activity of a child with low-functioning autism. It implicates the possiblity of applying DMBI as a rehabilitation program for patients with severe brain disorders.

##  Conflict of Interests

No competing financial interests exist.
